# The Decadence of the American Family

**Published:** 1882-08

**Authors:** 


### ARTICLE VIII.

### *The Decadence of the American Family.*

We do not like to be doleful, but it is impossible to ignore some of the facts that have been presented within the last year or two by Dr. Goodell, Dr. Nathan Allen, and others. These facts relate to an alleged change in the physical organization of our men and women, and to the decadence of family life among Americans. Dr. Allen, who has been studying this subject for many years, presents the case very directly in an article entitled "The New England Family" (*The New Englander*). It is asserted that the objects of the institution of the family are three: the propogation of children, the preservation of chastity, mutual help and company. In each of these respects the American family, especially the New England family, shows a marked and progressive deterioration, since one hundred years ago.

As regards the propagation of children, it is shown that the average native New England family is very much less productive than formerly. This, we are told, is due not alone to the induction of abortions and the prevention of conception, as was at one time asserted, but to a change in the physical organization of the parents. Individuals in whom the balance of tissues and functions is not well preserved are less fertile. The very nervous, or the very lymphatic temperaments accompany a lessened reproductive power. The tendency of the age is to a one-sided, intellectual cultivation and undue and artificial development of nervous tissue; hence a comparative sterility. The birth-rate in New England families has been steadily declining until now it is lower than that of any European country except France. One additional element in this, no doubt, is the habit of delaying marriages—a habit made almost necessary by the more expensive style of living which is demanded, and by what some consider the selfishness of young men who prefer not to sacrifice their liberty to the responsibility and expense of domestic life.

Another indication of family deterioration is the increase of divorces. These were rare a hundred years ago, now they number one to about twelve marriages in New England, while among our foreign populations, who are less educated, and even less moral, the ratio is one to forty or fifty.

Many evidences are brought forward to show that marriage does not accomplish all that it has done or ought to do in preventing adultery. The frequency of the charge of adultery in divorce suits is great, it being made in about one-third of the cases. The increase in late marriages, which leave many more of both sexes exposed to temptation, is an undoubted factor in the alleged increase of licentiousness.

As regards the third object of marriage, "mutual help and company," our author alleges the decrease in the number of marriages, the modes of life and business by which men are kept away from their homes, turning their whole energies in every direction except the making home pleasant. To the New York, as well as New England business man, home is often only a place to eat and catch his breath in during the intervals of business. It is also alleged that New England women are losing their love of offspring and home.

The family life of the American, therefore, according to the above indicment, occurs more rarely, begins later in life, is blessed with fewer offspring, is accompanied with less happiness and less fidelity; is, in fine, less of an institution than formerly. The more artificial attractions of this world have supplanted it.

Dr. Allen's remedy is in an attempt to revive the family spirit again. As the foundation of Church and State, as the institution by which good citizens are made, its preservation, he truly asserts, should be jealously guarded. The clergyman and moralist, the statesman and, by no means least, the physician are urged to use their influence to prevent this physical and family deterioration, which, although perhaps exaggerated by some, unquestionably exists.—*Editorial in Medical Record.*

